# Food entrainment: major and recent findings

**DOI:** 10.3389/fnbeh.2012.00083

**Published:** 2012-11-27

**Authors:** Breno T. S. Carneiro, John F. Araujo

**Affiliations:** ^1^Departamento de Ciências Animais, Universidade Federal Rural do Semi-ÁridoMossoró, Brazil; ^2^Programa de Pós-Graduação em Psicobiologia, Universidade Federal do Rio Grande do NorteNatal, Brazil; ^3^Departamento de Fisiologia, Centro de Biociências, Universidade Federal do Rio Grande do NorteNatal, Brazil

**Keywords:** circadian rhythm, food anticipatory activity (FAA), food entrained oscillator (FEO), scheduled feeding, neuronal activation

## Abstract

Mammals exhibit daily anticipatory activity to cycles of food availability. Studies on such food anticipatory activity (FAA) have been conducted mainly in nocturnal rodents. They have identified FAA as the behavioral output of a food entrained oscillator (FEO), separate of the known light entrained oscillator (LEO) located in the suprachiasmatic nucleus (SCN) of hypothalamus. Here we briefly review the main characteristics of FAA. Also, we present results on four topics of food anticipation: (1) possible input signals to FEO, (2) FEO substrate, (3) the importance of canonical clock genes for FAA, and (4) potential practical applications of scheduled feeding. This mini review is intended to introduce the subject of food entrainment to those unfamiliar with it but also present them with relevant new findings on the issue.

## Introduction

Circadian rhythms are ≈24 h oscillations in several domains (Kondo et al., 1993; Campos et al., 2001; DeCoursey, 2004) governed by circadian clocks, which are present in the central nervous system (CNS) and peripheral tissues of mammals (Dibner et al., 2010). Circadian clocks drive local rhythms so that biochemical and physiological processes occur at the appropriate time for optimal functioning of the organism. Peripheral and brain oscillators are synchronized indirectly to the alternating light–dark cycle by the hypothalamic suprachiasmatic nucleus (SCN) (Dibner et al., 2010). In the brain, circadian oscillators drive homeostatic and behavioral rhythms in a 24 h manner, in a way that feeding, drinking, and mating occur during the animal's active phase while fasting, and sleep occur in the animal's resting phase.

**Figure 1:**
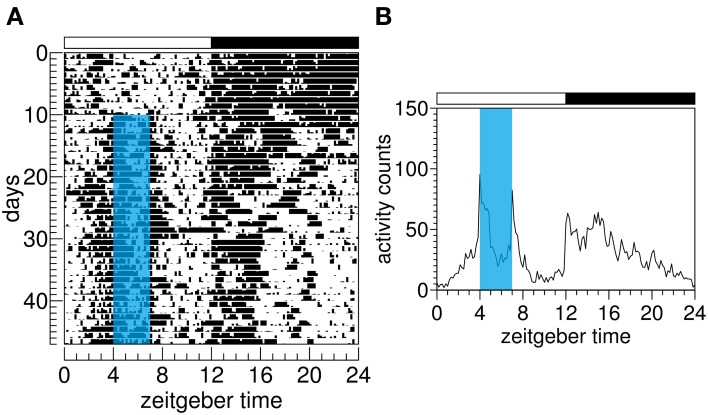
**Food anticipatory activity of one Wistar rat. (A)** Actogram shows the motor activity rhythm. In this type of representation, xy graphs (time/activity bouts) are plotted from the top to the bottom, representing subsequent days. Zeitgeber time refers to the time of the light–dark cycle. By convention, zeitgeber time 0 denotes light onset while zeitgeber time 12 denotes dark onset in a 12 h:12 h cycle. **(B)** Waveform depicts averaged activity over 37 days of scheduled feeding. Blue area on A and B indicates chow availability for 3 h each day. Food anticipatory activity is seen in both actogram and waveform graphs.

The light–dark cycle is the most potent *Zeitgeber* (environmental timing signal) for mammals; however, a range of non-photic signals is capable of entraining circadian rhythms. Cyclic food availability is one of them. Its effect on circadian rhythms has been mostly studied in rodents, but lagomorphs, marsupials, carnivores, and primates also entrain to scheduled food availability (O'Reilly et al., 1986; Zielinski, 1986; Boulos et al., 1989; Kennedy et al., 1990; Jilge, 1992; Juárez et al., 2012; Ware et al., 2012; Zhdanova et al., 2012). This non-photic entrainment is characterized by an increase in locomotion in the hours preceding food delivery. This anticipatory behavior was first reported in rats by Curt Paul Richter back in 1922 (Richter, 1922) and became known later as food anticipatory activity (FAA) (Figure 1).

Several experimental data show that FAA is mediated by a self-sustained circadian timing mechanism where a food entrained oscillator (FEO) is its principal component (reviewed in Mistlberger, 2009). Bolles and coworkers first showed that activity does not exhibit the expected rise in the 4 h before food availability in rats under 19 h or 29 h feeding (Bolles and de Lorge, 1962; Bolles and Stokes, 1965). This was the first evidence that FAA is absent if the length of the feeding cycle is too distant from 24 h, indicating a circadian limit on the system generating FAA. Other investigators later expanded evidence for the circadian nature of FAA by submitting rats to scheduled feeding at different periodicities (20–33 h) (Stephan, 1981; Mistlberger and Marchant, 1995).

One important aspect of FAA is its persistence in constant condition. For light entrained rhythms, constant condition means constant light or constant darkness. For FAA it means food deprivation. Bolles and Moot (1973) showed that food anticipatory behavior occurs during food deprivation tests in rats that had been previously fed with a meal schedule. Importantly, FAA occurred at the phase the animals had been feeding the days before. The persistence of FAA during total food deprivation was demonstrated later by Coleman et al. (1982). FAA persistence—in the absence of an entraining stimulus—implies that this phenomenon is generated by a self-sustained timing mechanism.

A report by Stephan and coworkers in 1979 that rats bearing SCN lesions could still anticipate cycles of food availability (Stephan et al., 1979) was one important evidence for the existence of, at least, two separate circadian systems in mammals: one entrained by light, that has the SCN as its principal component; and another entrained by food, which components are not fully characterized. Later reports showed that after phase shifting—advances or delays—the feeding time, FAA readjusted slowly to the new mealtime, presenting transients (days in which the anticipatory behavior occurs at a displaced phase, not aligned to feeding) (Stephan, 1984). This phenomenon indicates the existence of a gradual process by which the FEO is adjusted to feeding. This process involves daily phase shifts, which lead to entrainment of the FEO to feeding time. Phase shifts and entrainment for the FEO have, in principle, the same theoretical bases of phase shifts and entrainment of light entrained rhythms, which are very well-characterized (Refinetti, 2006).

The main research line on food entrainment has been the quest for the FEO. Also, some investigations have been done on possible input (external and internal) mechanisms to entrain the FEO (see Carneiro and Araujo, 2009). And more recently, the underlying molecular/genetic bases for FAA have been under investigation (Challet et al., 2009). In this paper, our purpose is to briefly review recent findings on four topics: (1) possible input signals to FEO, (2) FEO substrate, (3) the importance of canonical clock genes for FAA, and (4) potential practical applications of scheduled feeding.

## Input signals to the FEO

Recent studies have investigated the role of internal factors as timing signals for a brain-located FEO. Evidence points to such a configuration (Davidson et al., 2003). Then, assuming that the primary structures for FAA are in the brain, internal input signals derived from/associated with feeding should be necessary to tell the brain the time at which food is being available.

The possible role of humoral signals on the timing process of the FEO has been detailed elsewhere (see Carneiro and Araujo, 2009). Recent reports show attenuation but not extinction of FAA in mice deficient/impaired in ghrelin signaling (Blum et al., 2009; Verhagen et al., 2011). Blum et al. (2009) also reported, however, that chronic injection of ghrelin leads to a pattern of FOS expression in the arcuate nucleus (ARC) and dorsomedial nucleus of hypothalamus (DMH) similar to that seen in food restricted animals. In addition, ghrelin receptor knockout mice under scheduled feeding exhibit lower orexin expression and diminished neuronal activation of mesolimbic system (Lamont et al., 2012), which are recognized for their role on motivation (Calipari and España, 2012). These data suggest that despite of not being critical for FAA expression, ghrelin may be involved in the timing mechanism of brain areas entrained by food.

Another signal hypothesized of being involved on timing of the FEO is insulin (Carneiro and Araujo, 2009); however, data on this issue are scarce. Studying the plasma levels of insulin in the 90 min preceding food availability, Dailey et al. (2012) reported a close association of insulin levels and the timing of three meal schedules (chow, chocolate, and high-fat food). Insulin, besides controlling plasma glucose levels, is involved on control of food intake by acting on ARC (Cone, 2005). One earlier report revealed that insulin is unnecessary for food anticipation in diabetic transgenic rats (Davidson et al., 2002). Nevertheless, hypothesizing that the FEO is timed by more than one signal, insulin may be one of these timing elements.

## The arduous quest for the FEO

The search for a brain-located FEO started with studies of Krieger and Hauser (1977) and Stephan et al. (1979) in SCN lesioned rats maintained on a restricted feeding schedule. The results showed that the SCN was not the site of the FEO, since the rats still possessed the ability to anticipate timed food availability. The next years were of intense search for the FEO, with the brain lesion approach being the main methodology used. Basically, the investigation consisted of lesioning a specific area and submitting rats to scheduled restricted feeding. Several areas were tested to contain the FEO; however, the site of the oscillator was not identified (see Davidson, 2009). It is worth to mention studies on the role of the DMH. This issue has been under debate in recent years. Gooley et al. (2006) argued that DMH is critical for food anticipatory body temperature and activity rhythms in rats while Mistlberger and coworkers reported persistence of FAA in DMH-lesioned animals (Landry et al., 2006, 2007, 2011). The persistence of food entrained rhythms was confirmed later in DMH-lesioned mice (Moriya et al., 2009). Therefore, the DMH seems not to be uniquely responsible for FAA. However, recent papers indicate that this region may be important for disinhibition of behavioral expression (necessary for FAA) during the light phase, a function that would be accomplished by inhibition of neuronal activity in the SCN (Acosta-Galvan et al., 2011; Landry et al., 2011, see also Blum et al., 2012a for a discussion on this subject).

In the last decades, though, investigators started using markers for neuronal activity to identify cell populations that were activated/entrained/shifted by scheduled feeding. In the 2000's Escobar and coworkers showed that the pattern of c-fos expression in brainstem, hypothalamic and corticolimbic areas are shifted/altered by scheduled feeding (Ángeles-Castellanos et al., 2004, 2005, 2007). Other groups also have reported *c-fos*/FOS activation in many brainstem and hypothalamic nuclei in response to scheduled feeding (Meynard et al., 2005; Johnstone et al., 2006; Takase and Nogueira, 2008).

This approach revealed a spread effect of scheduled feeding on brain areas. This may have led to the consideration that the FEO is comprised of various sites, instead of a specific brain region, an idea discussed more systematically these days (Carneiro and Araujo, 2009; Mistlberger, 2011; Silver et al., 2011; Blum et al., 2012a).

Ribeiro et al. (2007) using a feeding phase shift protocol reported that the first hypothalamic neuronal population activated by restricted feeding is located in the ventromedial nucleus (VMH). The VMH is a sensor of fed/fast state and is involved in the control of food intake (King, 2006). There is evidence from lesion studies that the VMH is not solely responsible for FAA (Mistlberger and Rechtschaffen, 1984). However, this does not mean that this region does not contribute for FAA phenomenon in any way. Poulin and Timofeeva (2008), specifically found food anticipatory neuronal activation (*c-fos* mRNA) in anterior hippocampal continuation and the septohippocampal nucleus (AH/SHi), paraventricular nucleus of the thalamus (PVT) and DMH in rats. They also found that neuronal activation in parabrachial nucleus (PB) and nucleus of solitary tract (NTS) occurred only during feeding time. Recently, another structure, the cerebellum, was implicated in the regulation of food anticipatory rhythms (Mendoza et al., 2010). By monitoring clock gene expression in the cerebellum, these investigators demonstrated that this structure is sensitive to and phase shifted by scheduled feeding. In addition, lesion or circuitry alterations of Purkinje cells eliminated or strongly attenuated food anticipatory rhythms.

Recently, Blum et al. (2012b) used an unusual protocol to investigate neuronal activation (FOS) in response to scheduled feeding in mice. The animals were on a restricted feeding schedule for 14 days and sacrificed in the hour preceding feeding time. One group was sacrificed on 15th day while a second group after 3 days of *ad lib* feeding. The results show that ARC and DMH in the hypothalamus and NTS, the dorsal raphe (DR) and PB in the brainstem exhibit similar neuronal activation in the two conditions: (1) when mice are anticipating food during the feeding schedule and (2) when mice are no more anticipating food on *ad lib* condition. These data indicate persistent activation of these areas after termination of the feeding schedule and in the absence of FAA. The authors say that: *“… after a return to ad libitum conditions ONLY the regions that are necessary for timekeeping would remain preferentially activated”* (Blum et al., 2012b), which may point to a more relevant role of these regions as the neural substrate of the food entrained timing system.

## Clock genes and FEO mechanism

The identification of clock genes has led to the emergence of the field of molecular/genetic control of circadian rhythms. In mammals, the first clock gene implicated in the control of circadian rhythms, named *Clock*, was discovered by Vitaterna et al. (1994). Many other genes were identified as regulators of circadian rhythms and the molecular bases for function of circadian timing systems is fairly described (Lowrey and Takahashi, 2011).

Considering that known clock genes are responsible for generation of circadian rhythms at the molecular level, one plausible question is whether this molecular basis is also behind the generation of food entrained rhythms. Many investigators have tested the role of clock genes on FAA expression, mostly in mutant or knockout mice. Pitts et al. (2003) reported that *Clock* mutant mice are still capable of anticipating scheduled feeding. Dudley et al. (2003) showed soon after that NPAS2 (a *clock* paralog, expressed essentially in the forebrain, but not in the SCN, Zhou et al., 1997; Garcia et al., 2000) knockout mice also expressed FAA. In the actograms of these studies, we observe that, although still present, FAA seems to emerge late or to present a different duration (see Figure 3 in Pitts et al., 2003 and Figure 5D in Dudley et al., 2003). Storch and Weitz (2009) have investigated FAA in several clock genes-knockout mice. They reported daily FAA in *Per2*, *Per1/Per2*, and *Bmal1* knockout mice. Similar results were obtained by Pendergast et al. (2009) regarding *Bmal1* knockout mice. However, an earlier report showed that FAA was absent in *Per2*^*Brdm1*^ mutant mice (Feillet et al., 2006). This controversy is not fully understood but a gene mutation, creating a different protein, may generate different molecular interactions when compared with a complete knockout. *Clock* mutant mice present several metabolic alterations (Turek et al., 2005) and *Per2*^*Brdm1*^ mice show abnormal insulin function and glucose homeostasis (Carvas et al., 2012). These metabolic changes might contribute for changes in FAA pointed above.

Recently, Mieda and Sakurai (2011) reported that FAA emergence is delayed and persistence attenuated in conditional knockout *Bmal1* mice that lack *Bmal1* specifically in the CNS. Pendergast et al. (2012) reported that in *Per1/Per2/Per3* knockout mice, FAA is unstable and imprecise in a 24 h feeding schedule; yet, the animals are able to anticipate food. Interestingly, in addition to the 24 h rhythm, they exhibit a second period of ≈21 h. When put on a 21 h feeding schedule mice exhibit a more precise and stable food entrained rhythm. These results indicate that, though not critical for FAA expression (Storch and Weitz, 2009), *Per* genes may be involved in the molecular mechanism of FEO, at least in one aspect, period determination.

## Possible applications of feeding regimens

The SCN is not necessary for FAA, yet feeding regimens may affect SCN in some aspects. For example, there is evidence that caloric restriction affects SCN activity and circadian responses to light (Caldelas et al., 2005; Mendoza et al., 2005).

Two studies examined the effect of scheduled feeding on re-entrainment of motor activity after a light–dark phase shift (Ángeles-Castellanos et al., 2011; Carneiro and Araujo, 2011), reporting that rats under a feeding schedule re-entrain faster to the new LD cycle. Ángeles-Castellanos et al. (2011) showed that fast re-entrainment in scheduled fed animals is not due to a masking effect, but indeed is a result of an entrainment process. As the light entrained rhythm is controlled by the light entrained pacemaker in the SCN, the primary assumption is that, in this experimental condition, feeding may affect SCN by accelerating the re-entrainment process to LD. Potentially, similar protocols might be used to minimize the circadian alterations in people subjected to light–dark shifts or work schedules. To our knowledge, there is only one report of behavioral food anticipation in humans. By means of self experimentation, Roberts (2010) reported early awakening due to breakfast. He suggests this as anticipatory activity, similar to what animals exhibit. Of course, this result should be corroborated by more controlled studies with numerous subjects but it gives us an indication that behavioral rhythms in humans may be sensitive to scheduled food.

Sherman et al. (2011) reported that scheduled feeding decreases inflammatory and disease markers. Also, caloric restriction delays the onset of several diseases (Froy, 2011). It is hypothesized that robustness of peripheral rhythms and synchronization of biochemical, physiological, and hormonal rhythms lead to attenuation of ageing and consequently, the associated deterioration processes (Froy, 2011). The use of feeding regimens to affect such rhythms and circadian clocks may constitute an intervention tool for improving health conditions in the future.

## Final considerations

**Figure 2:**
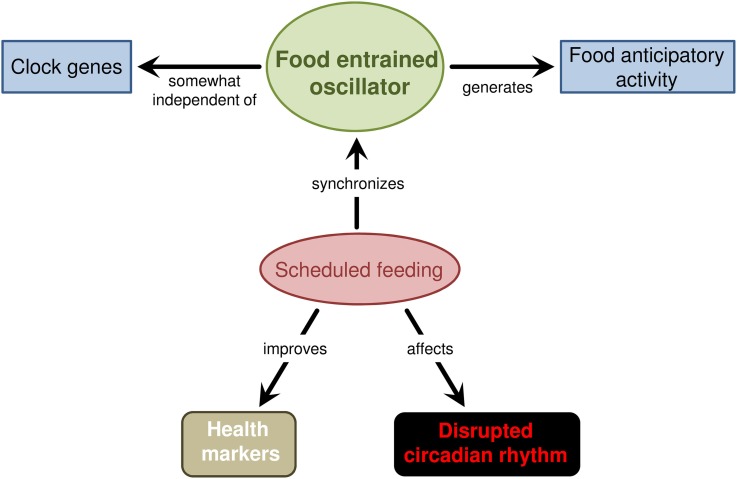
**A summarizing scheme on the effect of scheduled feeding in mammals.** These animals increase alert and locomotion before feeding time—named food anticipatory activity—when food is available at a specific moment of the day. This behavioral anticipation is generated by a food entrained oscillator that is synchronized by the recurring daily food availability. The anatomical organization of the food entrained oscillator remains elusive but the data indicate a system comprised of several structures, which in the brain may constitute local circuits in the hypothalamus, brainstem, and possibly other regions. Also, the FEO mechanism seems to be partially independent of the known clock genes since knockout or mutant mice rarely extinguish food anticipatory behavior. Scheduled feeding also seems to alleviate disrupted circadian rhythm and improve health.

FAA is observed when food is scheduled to a particular time of day. FAA is the behavioral output of the FEO in mammals. Despite continuous investigation in the past decades and identification of key characteristics of FAA, the anatomical organization of the FEO remains unsolved. Also, its molecular mechanisms are obscure and seem not to rely only on known clock genes (Figure 2).

Lesion studies indicated the possibility of a distributed organization (centrally/peripherally) of the FEO instead of discrete, and this alternative has been discussed more frequently in recent years (Mendoza et al., 2010; Mistlberger, 2011; Silver et al., 2011; Blum et al., 2012a). Mistlberger says that *“We could imagine instead a fully distributed food-entrainable clock system, in which oscillators in different brain regions and peripheral organs are entrained in parallel by feeding-related stimuli … ”* while Silver and coworkers state that *“These data indicate that the FEO timing system may engage a distributed circuit rather than a discrete localized site in the brain.”* We have proposed that such a central distributed FEO is entrained by fluctuation of various feeding-related humoral signals (Carneiro and Araujo, 2009). Yet, the input signals to the FEO remain undetermined.

Scheduled feeding has a broaden effect on brain activation. We believe that recent findings on neuronal activation are especially relevant for identification of structures comprising the FEO. These studies may be expanded by investigating the pattern of activation during the complete development of FAA and during food deprivation tests, in which FAA re-emerges.

Scheduled feeding also appears to act on disrupted circadian rhythm and health markers (Figure 2). Such effects may have practical applications on acute or chronic circadian disruption and human welfare.

### Conflict of interest statement

The authors declare that the research was conducted in the absence of any commercial or financial relationships that could be construed as a potential conflict of interest.
